# The Effect of the MgO/Al_2_O_3_ Ratio on the Thermal and Refractory Behaviors of Cordierite Ceramics

**DOI:** 10.3390/ma18010168

**Published:** 2025-01-03

**Authors:** Jae-Seung Lee, Jin-Woo Kim, Joo-Seok Park, Min-Ho Lee, Heesoo Lee

**Affiliations:** 1 Business Cooperation Center, Industry Support Division, Korea Institute of Ceramic Engineering & Technology, Jinju-si 52851, Republic of Korea; ljskdm9204@gmail.com (J.-S.L.); kimjw@kicet.re.kr (J.-W.K.); pjuju@kicet.re.kr (J.-S.P.); 2 School of Materials Science & Engineering, Pusan National University, Busan 46241, Republic of Korea

**Keywords:** cordierite, pyrometric cone, refractoriness, magnesium oxide, aluminum oxide, silicon oxide

## Abstract

In this study, cordierite-based ceramics (2MgO·2Al_2_O_3_·5SiO_2_) were synthesized using high-purity MgO, Al_2_O_3_, and SiO_2_ as starting materials. The influence of the MgO/Al_2_O_3_ ratio on various properties, including the thermal behavior, pyrometric cone refractory behavior, phase formation, physical properties, and microstructure of the synthesized ceramics, was systematically analyzed. Increasing the MgO/Al_2_O_3_ ratio progressively weakened the cordierite network, leading to lower temperatures for liquid formation and melting. This resulted in reduced viscosity and increased fluidity. Subsequently, the thermal and refractory behaviors were observed at lower temperatures with higher deformation rates under higher MgO/Al_2_O_3_ ratios. The lower viscosity of the liquid formed at reduced temperatures contributed to an increase in the density of sintered bodies, reduced porosity, and enhanced shrinkage. X-ray diffraction analysis confirmed that cordierite was the predominant phase in samples sintered at 1300, 1350, and 1400 °C, with higher cordierite formation at higher temperatures. Conversely, the formation of secondary phases, such as spinel, cristobalite, and enstatite, decreased with increasing sintering temperature. Pyrometric cones were then constructed for a range of temperature settings, and their deformation characteristics at specific temperatures were used to evaluate the refractoriness under diverse conditions.

## 1. Introduction

The ternary system of MgO–Al_2_O_3_–SiO_2_ contains cordierite as a crucial phase, as evidenced by its presence in the phase diagram of the system [1]. In this system, cordierite is in contact with six other phases—tridymite, enstatite, mullite, forsterite, spinel, and sapphirine—and achieves a balance with them [2]. This results in a narrow sintering temperature range of 1355–1460 °C [2,3], rendering sintering particularly challenging. Despite these challenges, cordierite has favorable properties, such as a low thermal expansion coefficient; a low dielectric constant; excellent thermal, mechanical, and electrical properties; and high thermal shock resistance [1,4,5,6,7,8]. These properties make it a suitable ceramic material for various industrial applications, including automotive honeycomb catalyst supports [9,10], ceramics [11], and refractories [12].

Various starting materials, such as polysiloxane [4,13], talc [6,14], kaolinite [1,2], kaolin [14,15], and clay [5,16], along with processes such as the sol–gel method [17,18], Pechini method [19,20], and glass-ceramic techniques involving high-temperature melting and crystallization [21,22], have been employed to synthesize cordierite. In this study, solid-state sintering was chosen for both stoichiometric and non-stoichiometric compositions of cordierite because it offers a straightforward approach that enables precise control over phase formation and the microstructure. This method not only simplifies the process but also ensures the production of ceramic bodies with cordierite as the major phase at a lower cost, making it particularly suitable for observing and analyzing the thermal and mechanical behaviors of the material. The addition of MgO in non-stoichiometric compositions is known to break existing Si–O–Si bonds within the silicate network, providing non-bridging oxygen (NBO), which weakens the network structure and reduces the viscosity [14]. Conversely, adding Al_2_O_3_ provides tetrahedral AlO_4_ units that strengthen the silicate network structure [21]. As confirmed through experiments, the structural differences in the silicate network due to the addition of MgO and Al_2_O_3_ influence the thermal behavior, refractory behavior, phase formation, physical properties, and microstructure of pyrometric cones.

In recent years, few studies have focused on the fabrication and behavior of pyrometric cones utilizing these properties. A pyrometric cone is a pointed, triangular-shaped specimen used to measure refractoriness, where the temperature at which the tip bends and touches the base indicates the refractory temperature. By conducting multiple tests with standard cones alongside the sample to be measured, the refractoriness of the sample can be determined by comparing it with the deformation temperature of the closest standard cone. Conventionally, pyrometric cones are made by combining ceramic raw materials such as feldspar, quartz, lime, and clay. However, the thermal properties of these standard cones can vary depending on the origin and condition of the raw materials. Therefore, it is necessary to prepare reliable pyrometric cones from high-purity raw materials for accurately measuring refractoriness.

Therefore, in this study, pyrometric cones based on cordierite were fabricated, and our results confirm that the refractoriness can be measured under various temperature conditions depending on the MgO/Al_2_O_3_ ratio.

## 2. Experimental Methods

### 2.1. Sample Preparation

Cordierite-based ceramics were fabricated using high-purity magnesium oxide (purity > 99.99%, Kojundo Chemical Laboratory Co., LTD., Sakado-shi, Japan), aluminum oxide (purity > 99.9%, Kojundo Chemical Laboratory Co., LTD., Sakado-shi, Japan), and silicon oxide (purity > 99.9%, Kojundo Chemical Laboratory Co., LTD., Sakado-shi, Japan) as starting materials. Based on the stoichiometric composition of cordierite, 2MgO:2Al_2_O_3_:5SiO_2_ (mass ratio: 13.7:34.9:51.4 wt%), the amount of SiO_2_ in moles was fixed, while the amounts of MgO and Al_2_O_3_ in moles were adjusted to gradually increase the MgO/Al_2_O_3_ (hereinafter referred to as M/A) ratio from samples C1 to C4. The compositions of all the samples fell within the cordierite region of the phase diagram (Figure S1); the specific compositions are presented in Table 1.

**Figure 1:**
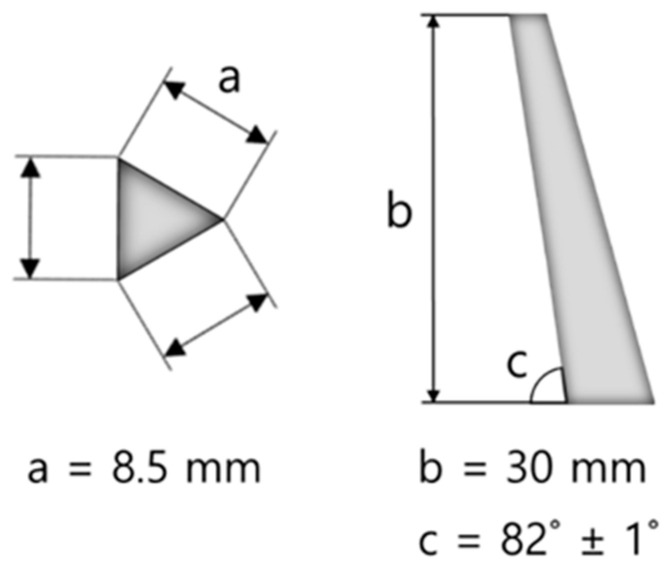
Standard cone dimensions and shapes.

The experimental workflow is summarized in Figure S2, outlining the processes of raw material preparation, ball milling, sieving, pressing, and sintering. Each step was carefully optimized to ensure the reproducibility and reliability of the cordierite-based ceramic samples for subsequent analysis. The raw materials (SiO_2_, Al_2_O_3_, MgO) were weighed according to the ratios specified in Table 1 and mixed with ethanol to form slurry. Zirconia balls (10 mm in diameter) were used to prevent any alteration of the composition. The volume ratio of the slurry to the zirconia balls was maintained at 1:1, and a dispersant (DS-203, SAN NOPCO, Pyeongtaek City, Republic of Korea) was added at 1 wt% based on the raw material to enhance mixing efficiency and prevent particle agglomeration. The milling process was conducted for 1 h to achieve uniform mixing. The speed was set at 64 rpm, which corresponds to 80% of the critical speed [23], to avoid centrifugation (where balls adhere to the wall of the container). After milling, ethanol was evaporated at 120 °C using a magnetic stirrer at 200 rpm. The dried powder was sieved through a 100-mesh screen to ensure consistent particle size distribution. The sieved powder was uniaxially pressed using a hydraulic press (Hydraulic Unit Model #3925, Carver, Inc., Indiana, USA) into two distinct shapes for different purposes. First, it was pressed into cylindrical shapes at a pressure of 100 MPa. These cylindrical samples were then sintered in an electric furnace (GSKF-15, KeSeong Scientific Co., Geumsan-gun, Republic of Korea) at three target temperatures: 1300, 1350, and 1400 °C, temperatures commonly used in the fabrication of refractory and cordierite ceramics [13,24]. To ensure accurate sintering temperatures, the electric furnace was calibrated up to 1600 °C (temperature accuracy of ±0.9 °C), enhancing the precision of the sintering process. At target temperatures exceeding 1400 °C, the samples failed during the 2 h holding period, indicating that temperatures beyond 1400 °C are unsuitable. Consequently, the optimal sintering temperature range for sample fabrication was determined to be 1300–1400 °C in this study. The heating rate was maintained at 10 °C/min, and the samples were held at the target temperature for 2 h. The sintering was conducted in an ambient air atmosphere, as widely utilized in previous experiments for the synthesis of cordierite ceramics [25]. After sintering, the samples were allowed to cool naturally to room temperature within the furnace [26]. This cooling method was chosen to minimize thermal shock and to ensure the phase stability of the sintered samples. Second, it was pressed at 100 MPa into a standard pyrometric cone shape using a mold designed according to the KS L ISO 1146 standard [27], as shown in Figure 1, and this was utilized to evaluate refractoriness.

### 2.2. Characterization

X-ray diffraction (XRD; SMARTLAB 3, Rigaku Co., Tokyo, Japan) analysis was conducted using CuKα radiation (*λ* = 1.5406 Å) at 40 kV and 30 mA. The scanning speed was set to 5°/min over a 2*θ* range of 7°–90°, and phase identification was performed using the ICDD database. Phase differences and peak variations between samples at different temperatures were compared by sintering at 1300, 1350, and 1400 °C for 2 h at the same heating rate. The density and porosity were measured using the Archimedes method in accordance with the KS L ISO 18754 [28], employing a 4-point balance (AX224KR, Ohaus Co., New Jersey, USA).. The linear shrinkage (%) was calculated by measuring the diameter change in the sintered cylindrical samples using a Vernier caliper (ABS Digimatic Caliper, Mitutoyo Co., Kawasaki-shi, Japan). A surface analysis of each sample sintered at 1400 °C was conducted using scanning electron microscopy (SEM; JSM-7610, JEOL, Tokyo, Japan) to compare microstructural differences.

The thermal behavior of the samples during heating was observed using a thermography camera, and all changes, including maximum sintering, softening, melting, and flowing states, were recorded. To ensure accurate temperature measurement, a calibrated electric furnace was used. However, as is well known, temperature deviations can occur within the furnace. Therefore, to minimize measurement errors, a thermocouple (MilliK, ISOTECH, Merseyside, UK) calibrated up to 1600 °C (temperature accuracy of ±0.003 °C) was placed near the samples to simultaneously measure the temperature. The temperature deviation between the furnace and the thermocouple was within ±0.8 °C. This approach was adopted as a reliable alternative to the conventional method using a high-temperature microscope, which may yield variable results depending on equipment conditions. By employing this method, sample deformation as the temperature increased was accurately recorded, along with the corresponding precise temperatures. The refractoriness test was conducted on samples molded into a standard cone shape (Figure 1), placed on refractory supports, and tested in accordance with KS L 3113 [29], KS L ISO 1146. To ensure accurate temperature measurement, the same conditions for the calibrated electric furnace and thermocouple were maintained, while a thermography camera was used to observe the refractory behavior and record all changes until the cone tip bent and touched the base.

## 3. Results and Discussion

The starting materials, MgO, Al_2_O_3_, and SiO_2_, were used to design compositions based on the stoichiometric formulation of cordierite, with increasing M/A ratios. The mixed raw materials were processed using a solid-state sintering method and fabricated into cylindrical samples. As described in Section 2.1, cylindrical samples sintered at 1300, 1350, and 1400 °C for 2 h were used to analyze phase formation, physical properties, and microstructural characteristics of the samples’ changes with sintering temperature. The thermal behavior was analyzed after only the pressing process was completed. Additionally, standard pyrometric cone-shaped samples, fabricated according to the KS L ISO 1146 standard, were utilized to analyze the differences in refractory behavior resulting from changes in the M/A ratio.

### 3.1. XRD Analysis

**Figure 2:**
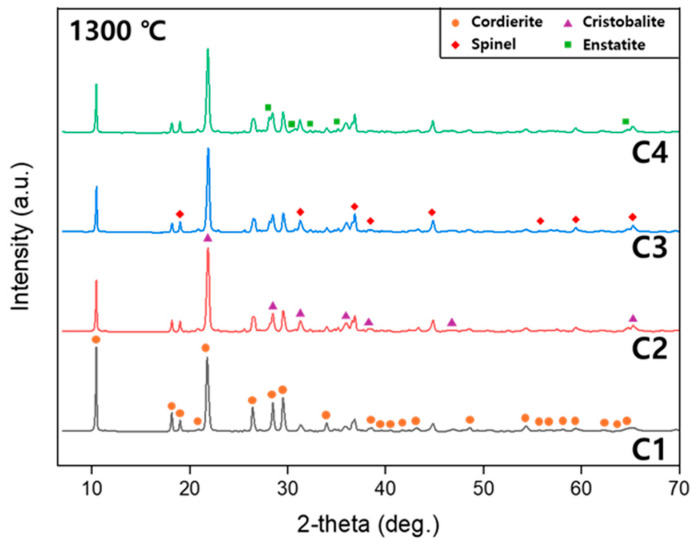

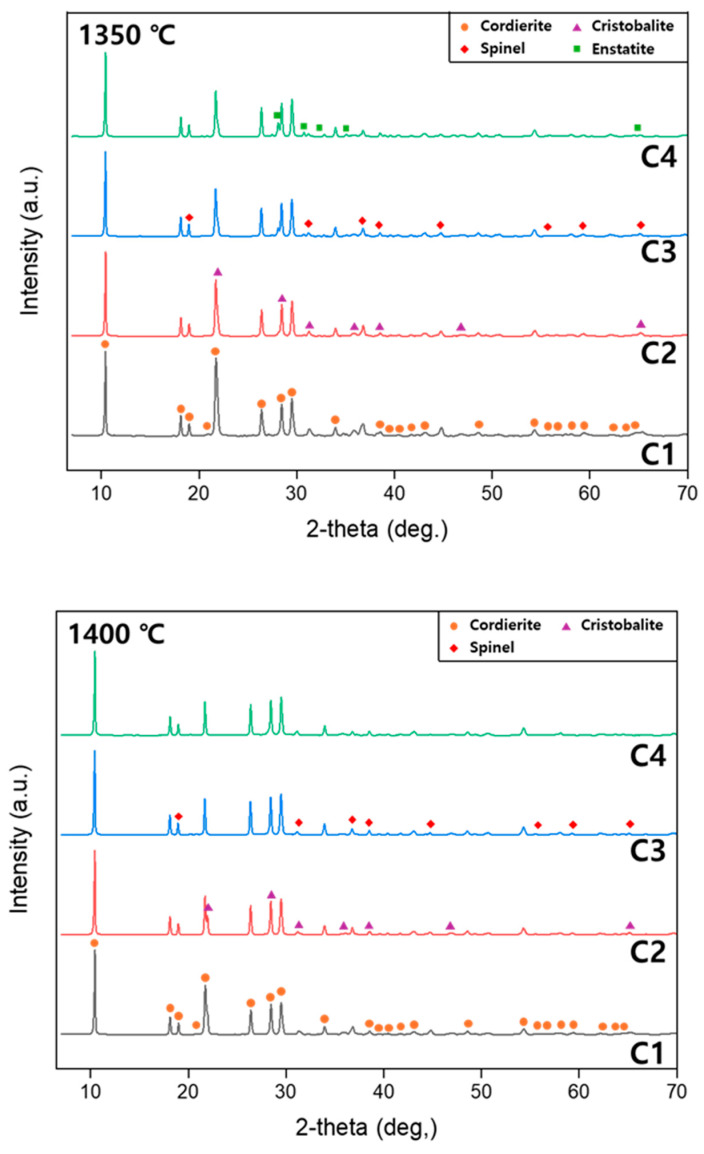
XRD patterns of the prepared ceramics at various sintering temperatures.

Figure 2 illustrates the crystal phases of samples C1 to C4 sintered at 1300, 1350, and 1400 °C for 2 h, as analyzed by XRD. Cordierite was observed as the primary phase in all sintered samples, with its intensity increasing as the sintering temperature rose. Additionally, the intensity of cordierite was highest in the stoichiometric composition (C1) and decreased as the M/A ratio increased, which also influenced the formation of secondary phases.

In the samples sintered at 1300 °C, cordierite (2MgO·2Al_2_O_3_·5SiO_2_, PDF 84-1220) was the primary phase, with cristobalite (SiO_2_, PDF 76-0937), spinel (MgAl_2_O_4_, PDF 77-0435), and enstatite (MgSiO_3_, PDF 11-0273) forming as secondary phases. Cristobalite formed by the crystallization of SiO_2_ during sintering [6,30]. Spinel formed through the interaction of AlO_6_ and MgO_6_ octahedral layers [2,6], where these layers, formed by the bonding of Al^3+^ and Mg^2+^ ions with oxygen ions at high temperatures, underwent mutual diffusion and rearrangement to create stable spinel. Enstatite is known to form through the reaction of MgO and SiO_2_ [31], and its intensity increased proportionally with the M/A ratio, being absent in the stoichiometric composition (C1) where the M/A ratio was the lowest. This result suggests that as the M/A ratio increased, the amount of Al_2_O_3_ decreased, leading to the reduced formation of cordierite and an increase in excess MgO and SiO_2_. In the samples sintered at 1350 °C, the intensity of cordierite increased, and cristobalite, spinel, and enstatite were observed as secondary phases. The intensity of cristobalite and spinel gradually decreased, likely because spinel reacts with cristobalite (SiO_2_) through mutual diffusion at high temperatures to form cordierite [2,30,32]. As the temperature exceeded 1300 °C, spinel and cristobalite were predicted to react and decrease while cordierite increases.

In the samples sintered at 1400 °C, cordierite exhibited the highest intensity, while the intensities of cristobalite and spinel decreased further. This is likely due to the increased reactivity at higher sintering temperatures, leading to more extensive reactions, forming cordierite. Additionally, enstatite was not observed, as it is known to react with excess Al_2_O_3_ and SiO_2_ at temperatures above 1300 °C to form cordierite [32]. Therefore, it is predicted that as the sintering temperature increases, Al_2_O_3_ and SiO_2_ will participate more actively in the reaction, resulting in increased cordierite formation.

### 3.2. Thermal Behavior During Heating

**Figure 3:**
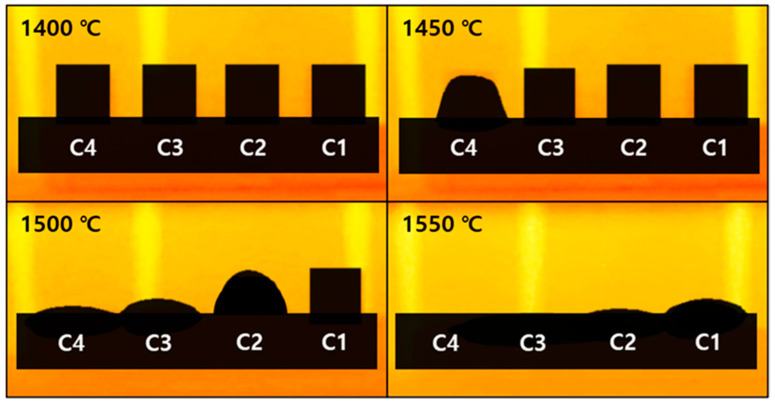
Thermal behavior of each sample at different temperatures.

To analyze the thermal behavior of ceramic samples, a calibrated electric furnace and a calibrated thermocouple were employed. These pieces of equipment ensured reliable and consistent temperature measurements and distribution within the furnace. Cylindrical samples (C1–C4), for which only the pressing process was completed, were placed inside the furnace with the thermocouple positioned near the samples. The samples were then heated at a constant rate of 10 °C/min until the target temperature was reached [33,34]. The thermal behavior of ceramic products can be observed through the sample deformation at high temperatures, which can be categorized into maximum sintering, initial softening, melting, and flowing temperatures based on the physical transformations observed [33]. To achieve this, a thermography camera was used, and all changes in the shapes of the samples and the corresponding temperatures throughout the entire heating process were comprehensively recorded, enabling the identification of these characteristic temperatures. This method ensured precise temperature control and consistent observations across different samples. Figure 3 illustrates the thermal behavior of samples with different M/A ratios, while a schematic diagram for better understanding is provided in Figure S3. As the temperature increased, the samples underwent densification and shrinkage during sintering. The maximum sintering temperature was defined as the point just before the onset of softening, at which the samples retained their angular edges [34]. At this point, the temperatures were recorded as C1: 1491 °C, C2: 1479 °C, C3: 1465 °C, and C4: 1441 °C. Beyond this temperature, softening occurs, causing the angular parts of the samples to gradually smoothen out and become rounded, indicating the onset of melting as a liquid phase forms on the surface [34,35]. As the temperature continued to rise, the samples transformed into a hemisphere shape with a height-to-base ratio of 1:2 [5,36]. At this stage, the corresponding temperatures were recorded as C1: 1539 °C, C2: 1500 °C, C3: 1484 °C, and C4: 1453 °C. Subsequently, complete liquefaction and flow were observed, with temperatures recorded as C1: 1553 °C, C2: 1512 °C, C3: 1491 °C, and C4: 1457 °C. Beyond these temperatures, no additional thermal behavior was observed; only flow occurred. It was confirmed that the characteristic temperatures of softening, melting, and flowing decrease as the M/A ratio increases.

**Figure 4:**
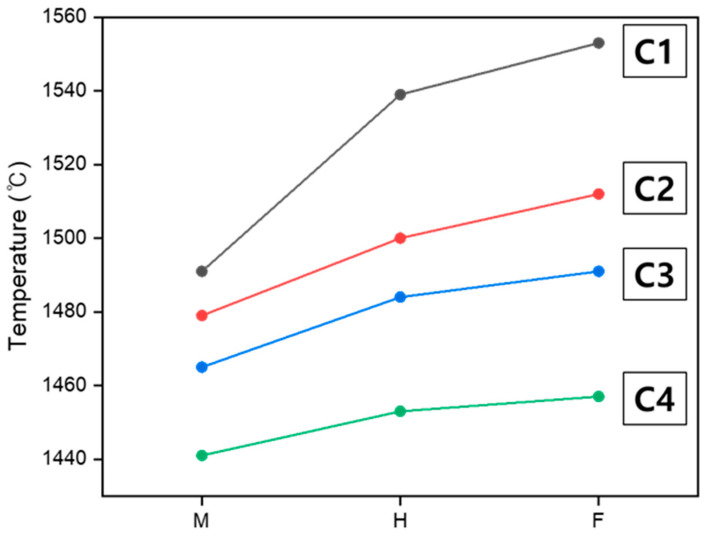
Changes in the maximum sintering temperature (M), hemisphere temperature (H), and flow temperature (F) in the various samples.

Cordierite is a silicate mineral characterized by its SiO_4_ tetrahedral structure, where a central Si^4+^ ion is surrounded by four O^2−^ ions. Each O^2−^ ion shares bonds with two Si^4+^ ions, repetitively linking silica tetrahedra to form a three-dimensional network structure [37]. Structural differences due to variations in Al_2_O_3_ content are known to affect the melting temperature and viscosity [21], and Al^3+^ ions act as network formers, demonstrating high efficiency in increasing the viscosity of the melt [38]. Al_2_O_3_ is introduced into the silicate network, forming Al^3+^ ions, which can participate in the network in a manner similar to Si^4+^ ions through tetrahedral coordination [37]. This forms tetrahedral groups by bonding with other SiO_4_ units, enhancing network connectivity and improving structural stability. To replace Si^4+^ ions with Al^3+^ ions, charge balance must be maintained, and as the Al_2_O_3_ content increases, Mg^2+^ ions can act as charge compensators, contributing to the charge balance of the network [37,39]. Meanwhile, the presence of MgO is known to increase the formation of NBO, which weakens the silicate network structure [14,21]. Mg^2+^, primarily derived from spinel (MgAl_2_O_4_), diffuses into the silicate network during high-temperature conditions. Mg^2+^ interacts with oxygen atoms, forming MgO_6_ octahedra that bridge SiO_4_ and AlO_4_ tetrahedra, contributing to the three-dimensional network structure. However, when excess Mg^2+^ is present, it disrupts the Si–O–Si bonds, replacing them with Mg–O–Si bonds, leading to the formation of NBO and weakening the network structure. Jiusti et al. reported that viscosity is a function of network connectivity, and the formation of NBO reduces network connectivity, thereby lowering the viscosity [40]. In this experiment, structural differences based on the M/A ratio were also found to affect the characteristic temperatures of softening, melting, and flowing. Additionally, the viscosity was influenced. The temperature difference between the maximum sintering state and the flow state was C1: 62 °C, C2: 33 °C, C3: 26 °C, and C4: 16 °C, with the time taken to reach the flow state from the maximum sintering state being C1: 6 min 12 s, C2: 3 min 17 s, C3: 2 min 36 s, and C4: 1 min 36 s. These results indicate that as the M/A ratio increases, the increased formation of NBO and the structural instability of the network not only reduce the characteristic temperatures of softening, melting, and flowing but also accelerate the thermal behavior because of the reduction in viscosity and the improvement in fluidity. Figure 4 shows that as the M/A ratio increases, the temperature difference between the maximum sintering temperature and the flow temperature decreases, and the slope of the temperature change becomes more gradual. This is attributed to the weakening of the network and the reduction in viscosity.

Figure 4 illustrates the changes in the maximum sintering temperature (M), hemisphere temperature (H), and flow temperature (F) for various samples with different M/A ratios. Each curve in the graph represents the trend in characteristic temperatures of softening, melting, and flowing:

M indicates the maximum sintering temperature, where the samples retain their angular edges.

H represents the temperature at which the samples transform into a hemisphere shape with a height-to-base ratio of 1:2.

F corresponds to the temperature at which complete liquefaction and flow occur.

As shown in Figure 4, the temperatures for M and F decrease with an increasing M/A ratio. Additionally, the temperature difference between M and F narrows, resulting in a more reduced slope. This trend reflects the weakening of the silicate network structure due to the increased formation of NBOs and the consequent reduction in network connectivity. These structural changes lead to a reduction in viscosity and an increase in fluidity, thereby accelerating the transitions between the characteristic temperatures of softening, melting, and flowing.

### 3.3. Physical Properties and Microstructure Analysis

**Figure 5:**
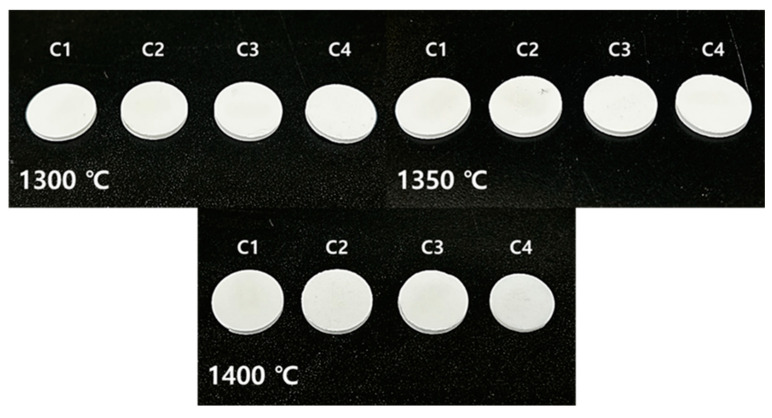
Shapes of cylindrical sample sintered at 1300, 1350, 1400 °C.

**Figure 6:**
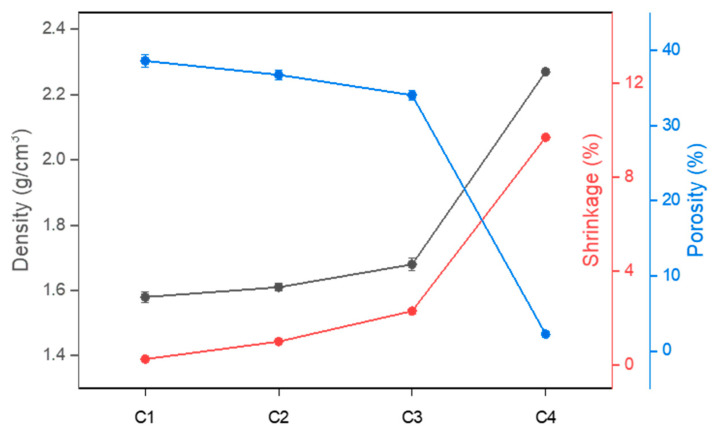
Density, porosity, and shrinkage rate of samples sintered at 1400 °C.

Figure 5 illustrates the shape of samples C1 to C4 after sintering at 1300, 1350, and 1400 °C. A comparison of the samples sintered at these temperatures shows that deformation of the sintered bodies according to the M/A ratio became evident at 1400 °C. The density of cordierite is known to be approximately 2.53 g/cm^3^ [7,16], and as shown in Figure 5, the greatest shrinkage occurred in C4, which exhibited a density closest to the theoretical density and the lowest porosity. Details are provided in Table 2. This result is interpreted as a consequence of liquid phase formation in each sample. At high temperatures, particles absorb thermal energy, which intensifies lattice vibrations and leads to bond breaking, resulting in the transition to a liquid phase at a certain temperature. As the M/A ratio increases, the liquid phase forms at lower temperatures, and the presence of NBO reduces the viscosity and increases the fluidity of the liquid phase. This behavior enhances mobility during the sintering process, enabling the liquid to easily infiltrate pores, thereby reducing porosity and promoting densification [41,42]. This process leads to the shrinkage of the samples (Figure 6) [30,41], resulting in higher density and lower porosity.

**Figure 7:**
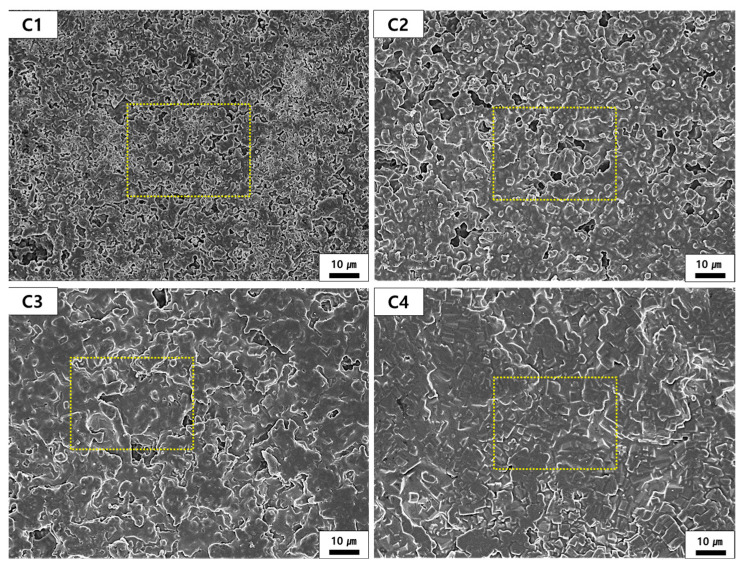
Microstructure (low magnification) of each sample sintered at 1400 °C.

**Figure 8:**
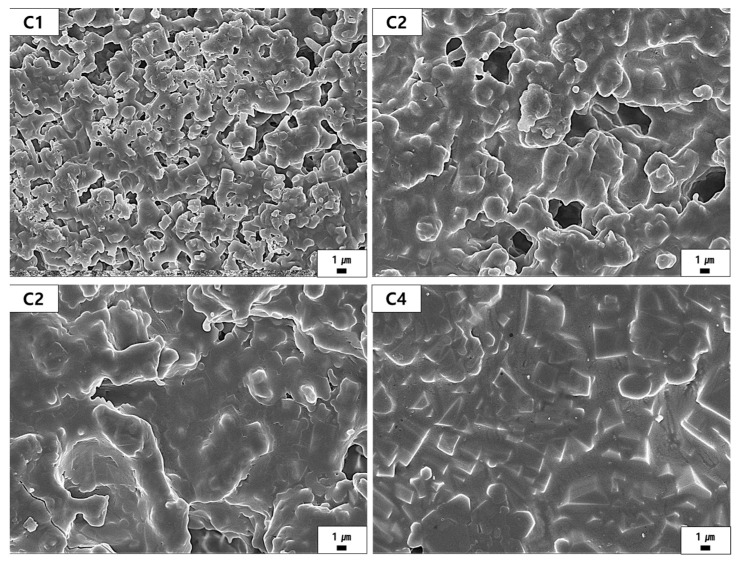
Microstructure (high magnification) of each sample sintered at 1400 °C.

The microstructure analysis presented in Figure 7 and Figure 8 illustrates the effects of the M/A ratio on the sintered samples. Figure 7 provides a low-magnification view of the overall microstructure, including the distribution of pores and the arrangement of grains, The yellow boxes in Figure 7 indicate selected regions for further high-magnification analysis in Figure 8, allowing detailed examination of typical microstructural features. Figure 8 reveals finer details, such as grain boundary diffusion, pore filling, and recrystallization patterns, which provide insights into the progressive densification and grain growth as the M/A ratio increases. These selected areas, while representative, were chosen to bridge the observations between low- and high-magnification analyses and ensure a comprehensive understanding of the microstructural evolution across the samples. As the M/A ratio increases, the number of visible pores decreases, and the structure becomes progressively denser. This trend can be attributed to the increased fluidity of the liquid phase formed during sintering, which promotes particle rearrangement and grain growth [21]. The liquid phase infiltrates between particles, enabling partial dissolution and freer movement, facilitating pore filling and the formation of a denser structure. As observed in Figure 8, these changes also contribute to grain agglomeration, where grain boundaries gradually blur, and recrystallization results in the growth of larger grains. At lower M/A ratios (e.g., C1 and C2), distinct grain boundaries and less pronounced grain growth are observed. In contrast, higher M/A ratios (e.g., C3 and C4) exhibit significant grain growth, leading to the formation of larger, polygonal grains [42]. Among the samples, C4 shows the most pronounced grain growth, corresponding to the highest fluidity of the liquid phase and the greatest reduction in pores. Additionally, EDS mapping was conducted to confirm the elemental distribution of Mg, Al, and Si in the C1 to C4 samples. The mapping results, as shown in Figure S4, indicate that these elements are uniformly distributed across the samples, suggesting that the sintering process was uniformly conducted.

### 3.4. Refractory Behavior of the Pyrometric Cone

**Figure 9:**
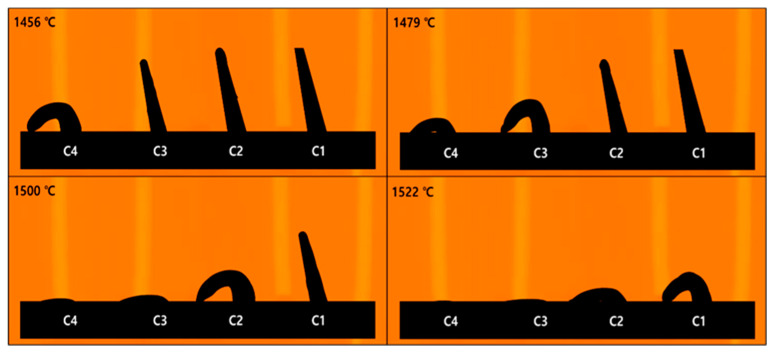
Refractoriness test using pyrometric cones.

**Figure 10:**
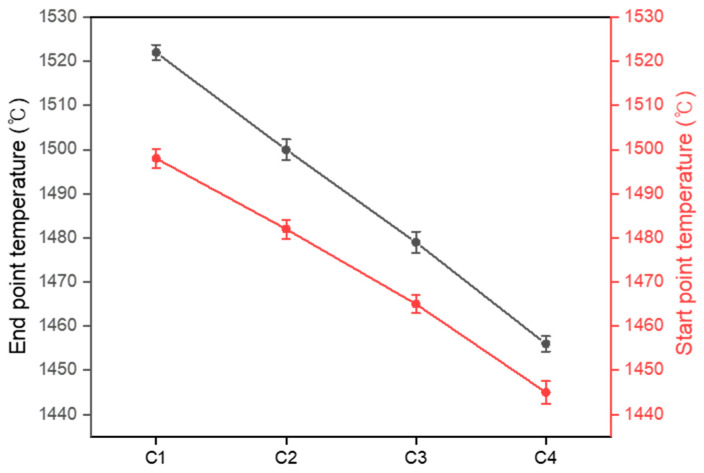
Refractoriness test results according to M/A ratio.

The refractoriness test was conducted according to standard test methods, starting at 1400 °C with a heating rate of 2.5 °C/min; the differences in the refractory behavior with respect to the M/A ratio are illustrated in Figure 9. The pyrometric cone, with its triangular shape, experiences a non-uniform temperature gradient as heat is transferred from the surface to the center; this causes the temperature to decrease toward the base of the cone, resulting in deformation beginning at the tip [43]. The solidus temperature, defined as the minimum temperature at which deformation begins, marks the onset of partial liquid formation [43]. This viscous liquid exhibits significant internal friction, necessitating greater force to induce flow, thereby indicating that strong interactions remain between the particles. As the temperature rises, viscosity gradually decreases, and the interactions between the particles begin to weaken. Hsieh experimentally demonstrated that deformation starts when viscosity reaches a certain critical value, with lower viscosity leading to deformation at lower temperatures [43]. The cone deforms according to the flow of the viscous liquid [44], with viscosity influencing the onset temperature and the rate of deformation. The average start point temperature at which the pyrometric cone began to deform was C1: 1498 °C, C2: 1482 °C, C3: 1465 °C, and C4: 1445 °C, and the average end point temperature at which deformation ceased was C1: 1522 °C, C2: 1500 °C, C3: 1479 °C, and C4: 1456 °C. The tests were run five times in total; the results of each test are shown in Table 3. The difference between the start and end point temperatures was C1: 24 °C, C2: 18 °C, C3: 14 °C, and C4: 11 °C, with the time taken from the start to the end of deformation being C1: 9 min 36 s, C2: 7 min 12 s, C3: 5 min 36 s, and C4: 4 min 24 s. As shown in Figure 10, these trends indicate that as the M/A ratio increased, both the temperature difference and deformation time decreased. From this result, it can be inferred that the differences in characteristic temperatures of softening, melting, and flowing, as well as the viscosity and fluidity influenced by structural differences, affect both the onset temperature and the rate of deformation. Consequently, as the M/A ratio increased, there was a reduction in deformation resistance and an acceleration in thermal behavior.

## 4. Conclusions

In this study, cordierite-based ceramics were fabricated using high-purity MgO, Al_2_O_3_, and SiO_2_ systems, and the effects of varying the M/A ratio on the thermal behavior, refractory behavior, phase formation, physical properties, and microstructure of pyrometric cones were systematically investigated. The following conclusions can be drawn.

1. As the M/A ratio increased from 1 to 1.86, the liquid formation temperature and the characteristic temperatures of softening, melting, and flowing decreased, the viscosity decreased, and the fluidity of the viscous liquid increased. The observed thermal behavior demonstrated that the maximum sintering temperature decreased from 1491 °C to 1441 °C, the hemisphere temperature decreased from 1539 °C to 1453 °C, and the flow temperature decreased from 1553 °C to 1457 °C. The time from the maximum sintering temperature to the flow temperature was reduced from 6 min 12 s to 1 min 36 s, indicating that the thermal behavior was accelerated. Additionally, the start point temperature of the pyrometric cone decreased from 1498 °C to 1445 °C, and the end point temperature decreased from 1522 °C to 1456 °C, with the time from the start to the end of deformation reduced from 9 min 36 s to 4 min 24 s, indicating an increase in the deformation rate.
2. As the M/A ratio increased from 1 to 1.86, a low-viscosity liquid phase was formed at lower temperatures, enabling it to easily infiltrate between particles. Consequently, the density of the sintered bodies increased from 1.56 g/cm^3^ to 2.27 g/cm^3^, the porosity decreased from 38.62% to 2.24%, and the shrinkage increased from 0.25% to 9.7%. As densification was promoted, grain boundaries gradually became blurred, and larger grains grew.
3. All the samples sintered at 1300, 1350, and 1400 °C contained cordierite as the primary phase, with more cordierite forming as the temperature increased. In contrast, secondary phases, such as spinel, cristobalite, and enstatite, tended to decrease as the sintering temperature increased. Additionally, it was observed that the intensity of cordierite decreased as the M/A ratio increased.

Our findings demonstrate how varying the contents of MgO and Al_2_O_3_ affects the thermal behavior, refractory properties, phase formation, physical properties, and microstructure of cordierite-based ceramics. By systematically investigating these factors, the research provides a comprehensive understanding of the influence of the M/A ratio on the properties of pyrometric cones prepared from cordierite.

## Figures and Tables

**Table 1:** Molar ratio of starting materials for cordierite synthesis and corresponding mass ratio (wt%) and MgO/Al_2_O_3_ ratio.

Sample	Mol Ratio	MgO	Al_2_O_3_	SiO_2_	MgO/Al_2_O_3_
**C1**	2:2:5	13.7	34.9	51.4	1
**C2**	2.2:1.8:5	15.4	32.1	52.5	1.22
**C3**	2.4:1.6:5	17.2	29.2	53.6	1.5
**C4**	2.6:1.4:5	19.0	26.1	54.9	1.86

**Table 2:** Density, porosity, and shrinkage rate of samples sintered at 1400 °C.

Parameter	C1	C2	C3	C4
**Density (g/cm^3^)**	1.56	1.59	1.65	2.27
**Porosity (%)**	38.62	36.75	34.04	2.24
**Shrinkage (%)**	0.25	1	2.3	9.7

**Table 3:** Start point temperature and end point temperature (°C) of each sample.

Sample	C1	C2	C3	C4
**Test 1**	1498/1525	1483/1498	1462 /1481	1442/1453
**Test 2**	1495/1523	1481/1497	1468 /1483	1449/1457
**Test 3**	1500/1521	1485/1501	1465/1479	1446/1455
**Test 4**	1501/1520	1479/1503	1463/1476	1443/1458
**Test 5**	1497/1523	1480/1502	1465/1478	1447/1457

## Data Availability

The original contributions presented in the study are included in the article/Supplementary Materials, further inquiries can be directed to the corresponding authors.

## Supplementary Materials

The following supporting information can be downloaded at: https://www.mdpi.com/article/10.3390/ma18010168/s1, Figure S1: Compositional ratios within the crystallization region of cordierite. Figure S2: Experimental workflow: (a) raw material preparation process (b) forming and sintering process. Figure S3: Schematic representation of the thermal behavior of samples at elevated temperatures: (a) the initial state with no deformation, (b) maximum sintering, (c) the onset of softening, (d) the formation of a hemisphere, and (e) transition to a fully flowing state. Figure S4: EDS mapping of Mg, Al, and Si in each sample at 1400 °C.
