# Supplementary material for: The Effect of the MgO/Al2O3 Ratio on the Thermal and Refractory Behaviors of Cordierite Ceramics
Source: Materials (Basel). 2025 Jan 3;18(1):168. doi: 10.3390/ma18010168 (PMC11721456; doi:10.3390/ma18010168)
Supplement: Supplementary file 1 [file materials-18-00168-s001.zip › materials-3355618-supplementary.pdf]

Supplementary Information

Effect of MgO/Al_2O_3 Ratio on the Thermal and Refractory Behaviors of Cordierite Ceramics

Jae-seung Lee [1, 2], Jin-woo Kim [1], Joo-Seok Park [1], Min-Ho Lee [1, 2*], Heesoo Lee [2*]

[1] Business Cooperation Center, Industry Support Division, Korea Institute of Ceramic Engineering & Technology, Jinju-si, Republic of Korea;

[2] School of Materials Science & Engineering, Pusan National University, Busan 46241, Republic of Korea; heesoo@pusan.ac.kr

* Correspondence: heesoo@pusan.ac.kr (H.S.L.)

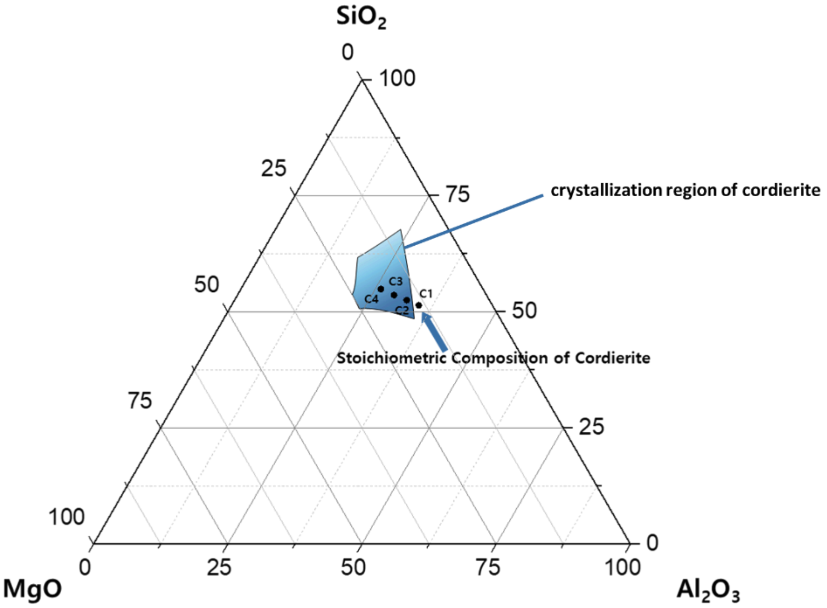

Figure S1. Compositional ratios within the crystallization region of cordierite.

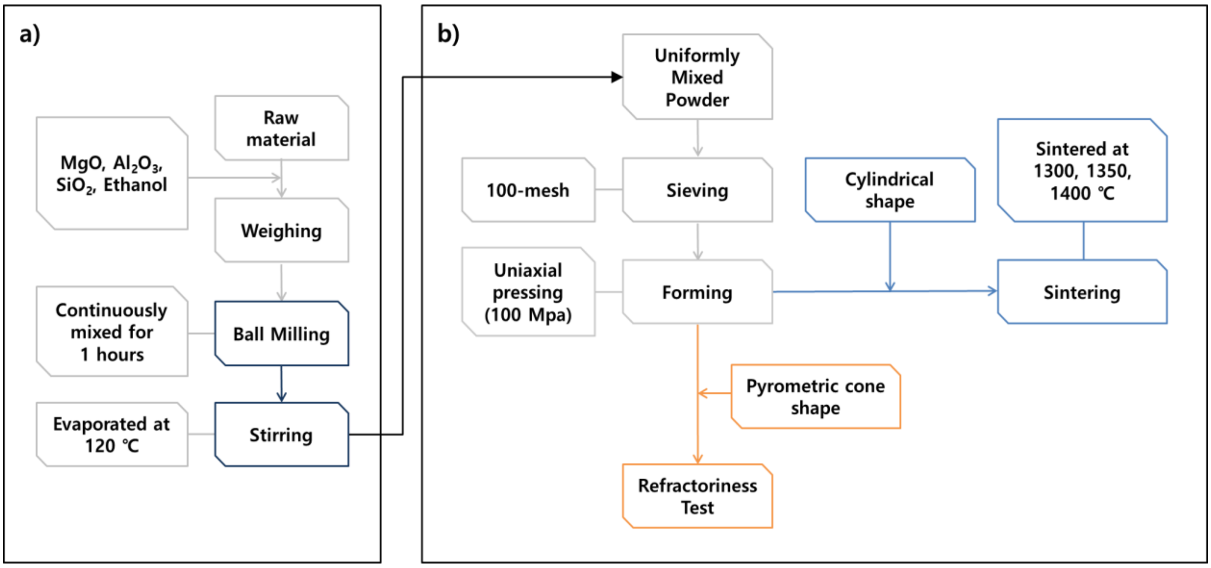

Figure S2. Experimental workflow: a) raw material preparation process b) forming and sintering process.

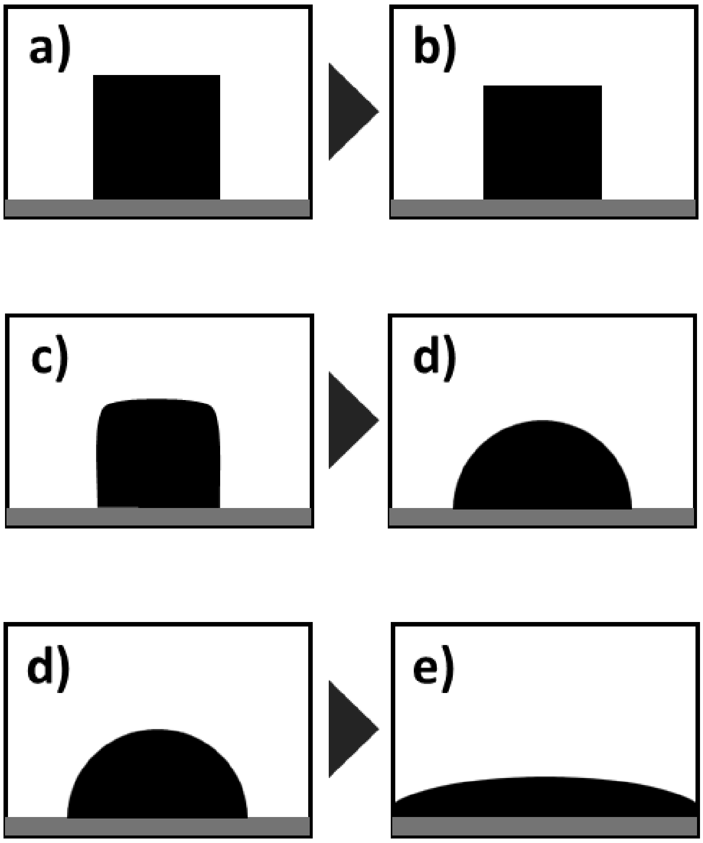

Figure S3. Schematic representation of the deformation behavior of samples at elevated temperatures: (a) the initial state with no deformation, (b) maximum sintering, (c) the onset of softening, (d) the formation of a hemisphere, and (e) transition to a fully flowing state.

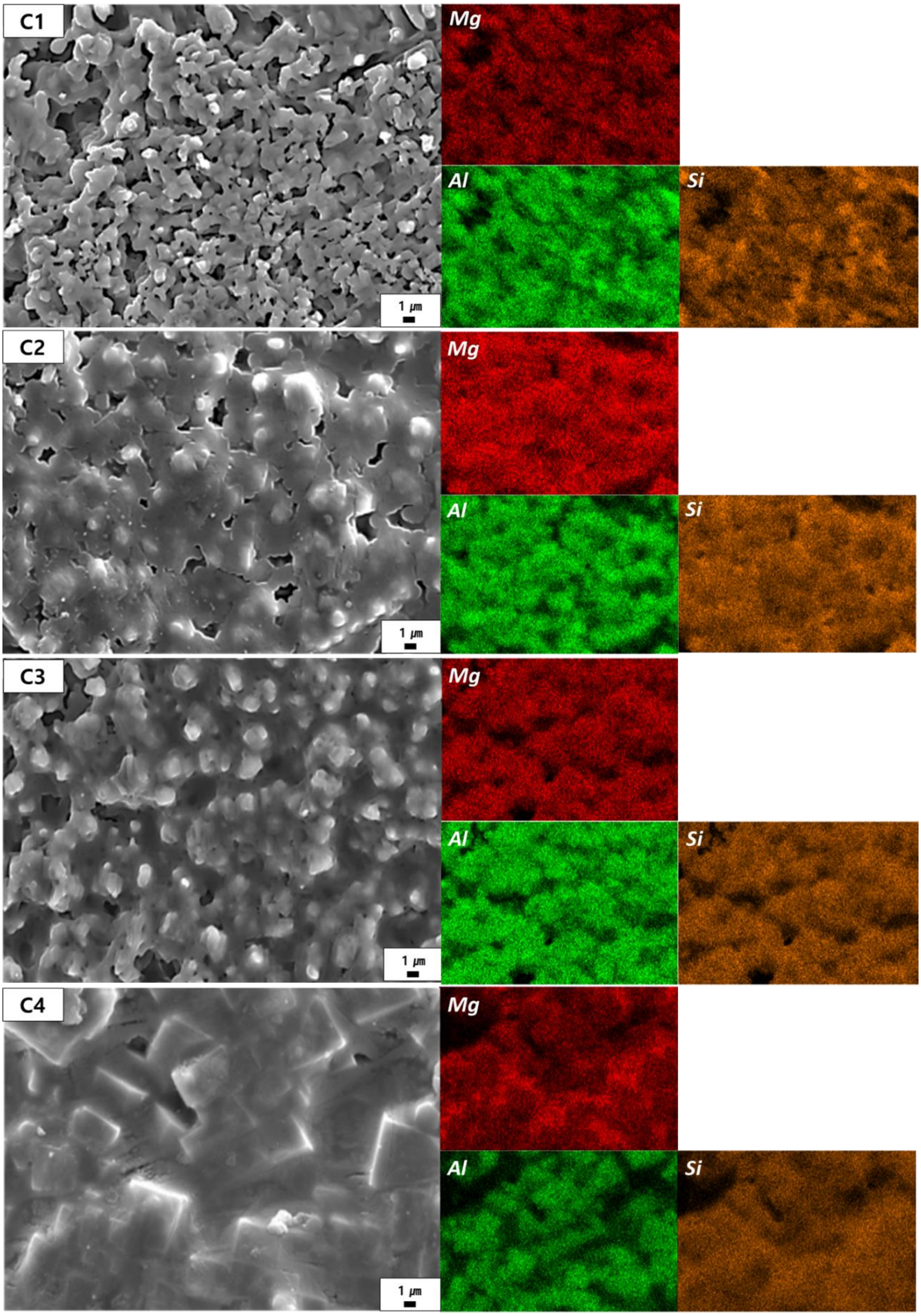

Figure S4. EDS mapping of Mg, Al, and Si in each sample at 1400 °C.
